# Improved early postoperative cognition in elderly gastrointestinal patients: a randomized controlled trial on the role of ultrasound-guided stellate ganglion block

**DOI:** 10.3389/fnagi.2025.1503314

**Published:** 2025-02-11

**Authors:** Ruyue Xue, Yuexian Li, Mei Zhan, Lin Yang, Defeng Sun

**Affiliations:** Department of Anesthesiology, First Affiliated Hospital of Dalian Medical University, Dalian, China

**Keywords:** stellate ganglion block, gastrointestinal surgery, postoperative cognitive dysfunction, oxidative stress, inflammation

## Abstract

**Background:**

This study evaluates the impact of ultrasound-guided stellate ganglion block (SGB) on early postoperative cognitive dysfunction (POCD) in elderly patients who underwent laparoscopic gastrointestinal (GI) surgery, as well as its potential effect on oxidative stress and inflammatory responses.

**Methods:**

In this randomized controlled trial, 104 elderly patients scheduled for elective laparoscopic GI surgery were randomized to receive ultrasound-guided SGB before general anesthesia (SGB group) or general anesthesia alone (control group). A total of 98 patients completed the study. Cognitive function was assessed using the Mini-Mental State Examination (MMSE) and Montreal Cognitive Assessment (MoCA) preoperatively, and on postoperative days one and three. The perioperative recordings included mean arterial pressure, heart rate, and the bispectral index. Blood samples were analyzed for interleukin-6 (IL-6), superoxide dismutase (SOD), and malondialdehyde (MDA).

**Results:**

The SGB group had a significantly lower incidence of POCD on postoperative day one (*p* < 0.05). IL-6 and MDA levels were significantly lower, while SOD levels were higher in the SGB group, when compared to the control group (*p* < 0.05). MDA levels were notably lower on postoperative day three in the SGB group (*p* < 0.05). Both groups showed significant changes in IL-6, SOD and MDA levels, when compared to preoperative values. The hemodynamic indicators showed a slight reduction in intraoperative blood pressure and decreased numerical rating scale scores on the first postoperative day without significant differences in other indicators.

**Conclusion:**

Preoperative SGB reduces early POCD in elderly patients who undergo laparoscopic GI surgery, possibly through the inhibition of oxidative stress and inflammatory responses.

## 1 Introduction

The global trend of an aging population, combined with advances in medical care, has resulted in a significant increase in the number of elderly individuals undergoing surgical procedures (Gou et al., 2021). This is particularly pronounced in China, where gastrointestinal (GI) diseases are highly prevalent among the elderly population, posing a significant threat to life expectancy and necessitating a corresponding increase in surgical procedures (GBD 2019 Diseases and Injuries Collaborators, 2020; Saur et al., 2021). This trend underscores the critical need for specialized surgical care and customized postoperative management strategies for the elderly demographic. A major concern is postoperative cognitive dysfunction (POCD), which is a commonly observed complication marked by a significant decline in cognitive functions, such as memory, attention, and executive function.

The incidence of POCD varies widely, with reports indicating a prevalence that range within 10–50% following general surgery. Age is considered an independent risk factor for the development of POCD (Kim et al., 2016). For elderly patients who underwent complex or emergency procedures, the incidence was even higher, reaching up to 17–61% (Chen J. et al., 2022). For instance, the incidence of POCD following major non-cardiac surgery in elderly patients was reported to be 25.8% (Zeng et al., 2023). Among elderly patients who underwent colorectal surgery, POCD occurred in 8.2–54% of cases (Mosk et al., 2018). Specifically, in elderly gastric cancer patients who underwent laparoscopic gastrectomy, 27.4% experienced POCD within seven days, postoperatively (Chen J. et al., 2022). This high prevalence underscores the profound impact of POCD on patient recovery, leading to increased risks of prolonged hospitalization, long-term disability, and diminished quality of life (Lin et al., 2020).

Although the prevalence of POCD in elderly surgical patients has been well-documented, its complex and multifactorial etiology remains poorly understood, thereby hindering the development of effective treatments. Factors, such as anesthesia, surgical trauma, inflammation, and underlying neurodegenerative diseases, have been considered to influence POCD (Lin et al., 2020). Despite the substantial impact on patient outcomes and healthcare costs, effective therapeutic strategies for POCD remains lacking. This highlights the urgent need for continued research into its underlying mechanisms, and the exploration of potential interventions to mitigate its effects in elderly surgical populations.

Emerging research has highlighted the critical role of neuroinflammation and oxidative stress in the pathogenesis of POCD. Specifically, microglial activation, the IL-6 trans-signaling pathway, and blood–brain barrier (BBB) disruption have been implicated, highlighting the need for a deeper mechanistic understanding (Skvarc et al., 2018; Lin et al., 2020; Li et al., 2022). Clinical evidence further supports these findings, demonstrating a strong correlation between systemic inflammatory markers, notably IL-6, and the severity of POCD in elderly patients underwent GI surgery (Barreto Chang and Maze, 2022). Furthermore, research has revealed an increase in oxidative markers, such as MDA, and a decrease in antioxidant capacities, including SOD, in patients with POCD (Su et al., 2020), indicating a critical imbalance between oxidative and antioxidative systems. These insights collectively highlight inflammation and oxidative stress as potential therapeutic targets for mitigating POCD (Ho et al., 2020).

Recent studies have suggested that ultrasound-guided stellate ganglion block (SGB), which is a minimally invasive technique that targets the cervical sympathetic nervous system, holds promise for mitigating POCD in elderly patients who undergo GI surgery. Ultrasound-guided SGB has been shown to reduce POCD incidence, possibly by modulating surgical stress responses, and promoting postoperative recovery (Deng X. et al., 2023). In addition, ultrasound-guided SGB has demonstrated the potential to enhance hemodynamic stability, and improve POCD outcomes (Sun et al., 2024). Although the precise mechanisms underlying these effects remain unclear, animal studies have suggested that SGB may exert its protective effects through anti-inflammatory and antioxidant mechanisms (Yu et al., 2023). Furthermore, despite these promising findings, the impact of ultrasound-guided SGB on POCD in elderly patients who underwent GI surgery remains not fully understood, highlighting the need for further research to elucidate its efficacy and underlying mechanisms in this specific patient population.

The present study aims to address this critical knowledge gap by determining the effects of preoperative ultrasound-guided SGB on the incidence and severity of POCD in elderly patients undergoing GI surgery. The investigators hypothesize that SGB can effectively prevent POCD in this population. The present study will contribute to a better understanding of the potential benefits of SGB in preventing POCD, and improving postoperative outcomes in this vulnerable population.

## 2 Materials and methods

### 2.1 Study design and participants

The present study was approved by the Ethics Committee of Dalian Medical University (PJ-KS-KY-2023-168[X]), and the trial was registered prior to patient enrollment at China Clinical Trial Center (ChiCTR2300075264, Principal investigator: Ruyue Xue, Date of registration: 31/08/2023). Although preliminary recruitment activities, including pilot testing, began in June 2023, formal enrollment and data collection for the registered trial commenced after registration in August 2023. Furthermore, the present study enrolled 104 patients who were 65 years old or older, and were scheduled for elective laparoscopic GI surgery under general anesthesia at the First Affiliated Hospital of Dalian Medical University, between June and December 2023.

#### 2.1.1 Inclusion criteria

Patients were eligible for inclusion when they were 65 years old or older, had an American Society of Anesthesiologists (ASA) physical status classification of I-III, and underwent laparoscopic GI surgery with an anticipated duration of two hours or greater. The additional inclusion criteria were as follows: body mass index (BMI) of ≤30 kg/m^2^ (Kitsis et al., 2022), preoperative MoCA score of ≥26, and preoperative MMSE score of >23. All participants provided a written informed consent prior to enrollment.

#### 2.1.2 Exclusion criteria

Patients were excluded when they presented with any of the following: contraindications to SGB; history of neurological or psychiatric disorders, including neurodegenerative diseases; recent use of sedatives, analgesics, anticholinergic drugs, antidepressants, corticosteroids, or other immunosuppressive medications; coagulation disorders; liver or kidney dysfunction (Child-Pugh C class, serum creatinine >125 μmoL/L).

#### 2.1.3 Randomization and blinding

Eligible patients were randomized at a 1:1 ratio to the SGB group or control group using a computer-generated random number generator. Patients in the SGB group received 4 mL of 0.25% ropivacaine *via* ultrasound-guided SGB, while patients in the control group received an equal volume of normal saline using the same technique.

### 2.2 Anesthesia monitoring and SGB procedure

Upon entering the operating room, intravenous access was established, and standard anesthetic monitoring, which included electrocardiography (ECG), non-invasive blood pressure (NIBP) monitoring, heart rate (HR) monitoring, pulse oximetry (SpO_2_), bispectral index (BIS) monitoring, and body temperature (T) monitoring, was initiated.

Patients who were randomized to the SGB group underwent ultrasound-guided SGB prior to the induction of general anesthesia. Briefly, the patient was placed in the supine position with a thin pillow under the right shoulder to facilitate optimal neck exposure. After skin disinfection, a high-frequency linear array transducer (6–13 MHz) was positioned at a 45-degree angle to the patient’s sagittal plane to identify the transverse process of the sixth cervical vertebra (C6) and adjacent anatomical structures under ultrasound guidance. Then, an in-plane approach was used for needle advancement. Since the stellate ganglion is often not clearly visualized, 4 mL of 0.25% ropivacaine was injected into the adjacent longus colli muscle. The successful blockade was confirmed by the development of Horner’s syndrome on the right side, which is characterized by miosis, ptosis, facial anhidrosis, and conjunctival injection.

After the SGB or sham procedure, a right radial artery catheter and right internal jugular vein catheter were placed under ultrasound guidance using local anesthesia for continuous intraoperative hemodynamic monitoring and intravenous medication administration.

### 2.3 Anesthesia management

General anesthesia was induced with intravenous propofol (1–2 mg/kg), butorphanol (40 μg/kg), and cisatracurium (0.15 mg/kg). After the confirmation of adequate neuromuscular blockade, the trachea was intubated, and the lungs were mechanically ventilated. The mechanical ventilation settings were, as follows: tidal volume of 6–8 mL/kg of predicted body weight, respiratory rate of 12–14 breaths/min, and inspired oxygen concentration of 50%, with an end-tidal carbon dioxide partial pressure target of 35–40 mmHg.

Anesthesia was maintained with a combination of intravenous propofol (2–3 mg/kg/h) and cisatracurium (0.1–0.12 mg/kg/h) infusions, and inhaled sevoflurane (1–2%). Additional doses of intravenous butorphanol were administered as needed, based on the patient’s response, in order to maintain adequate analgesia.

At approximately 30 min prior to the anticipated end of surgery, the cisatracurium infusion was discontinued. Upon surgical completion, the propofol infusion was stopped, and a patient-controlled intravenous analgesia (PCIA) pump was initiated for postoperative pain management. The PCIA solution comprised butorphanol (0.15 mg/kg, total dose) diluted in 100 mL of normal saline. Then, the patients were transferred to the post-anesthesia care unit (PACU).

Tracheal extubation was performed when the standard clinical extubation criteria were met. After extubation, the patients were discharged from the PACU to the surgical ward when they achieved a modified Aldrete score of ≥12, with no individual score of <1.

### 2.4 Postoperative care and data collection

Postoperative care was managed by a dedicated anesthesiologist. On postoperative days one (T8) and three (T9), the patients underwent comprehensive assessments, which included pain evaluation using the numerical rating scale (NRS) and the documentation of any postoperative nausea and vomiting (PONV) events. Cognitive function was evaluated by a psychiatrist on T8 and T9 using both the MMSE and MoCA. Postoperative cognitive function decline was defined as having a Z-score of ≤ − 1.96 on both the MMSE and MoCA, with scores on both tests ranging from 0 to 30.

The present prospective study meticulously documented several key parameters. The baseline patient demographics were initially recorded. Subsequently, hemodynamic monitoring was implemented, which comprised of the mean arterial pressure (MAP), HR, and BIS. These parameters were systematically recorded at six distinct time points: prior to SGB block placement (T1), preceding anesthetic induction (T2), at the induction of anesthesia (T3), at five minutes post-induction (T4), at the commencement of surgery (T5), and at five minutes following surgical incision (T6).

Total surgical duration was defined as the interval between skin incision and final suture placement. Similarly, the total anesthesia duration spanned from the initiation of general anesthesia to its cessation. Furthermore, the study meticulously monitored the consumption of four anesthetic agents: propofol, cisatracurium, butorphanol, and methoxamine hydrochloride. Lastly, the incidence of postoperative complications, notably pain and PONV, was diligently recorded.

### 2.5 Oxidative stress and inflammatory markers detection

Peripheral venous blood samples (3 mL) were collected from the non-infusion arm at four distinct time points: one day before surgery (T0), immediately following PACU discharge (T7), on the first postoperative day (T8), and on the third postoperative day (T9). The collected samples were placed in ethylenediaminetetraacetic acid (EDTA)-coated tubes, and centrifuged at 3,000 rpm for 10 min. Then, the supernatant was collected and stored at −80°C until analysis. The serum levels of IL-6, SOD and MDA were measured using commercially available enzyme-linked immunosorbent assay (ELISA) kits (Kexing Biotech, Shanghai, China).

### 2.6 Sample size calculation

Based on the review of relevant literature and pilot studies, the incidence of POCD in elderly patients with GI tumors, who underwent laparoscopic surgery, was approximately 35% (Chen X. et al., 2022). SGB has been shown to reduce the incidence of POCD to 10.70%. Using a two-sided *t*-test with a power of 80% and a significance level of 0.05, a sample size of 46 patients per group was determined using the PASS 15.0 software (NCSS, East Kaysville, Utah, USA). Assuming a 10% dropout rate, the required final sample size was 52 patients per group, for a total of 104 patients.

### 2.7 Statistical analysis

The statistical analysis was conducted using SPSS version 26.0 (IBM Corp, Armonk, NY, USA). Continuous variables were initially assessed for normality. Normally distributed data were presented in mean ± standard deviation (SD), while non-normally distributed data were presented in median (M) and interquartile range (IQR). Categorical variables were presented in frequency (%).

The Mann–Whitney *U*-test was employed to compare non-normally distributed continuous variables between groups, while Student’s *t*-test was used for normally distributed continuous variables. Categorical variables were compared using Chi-square test or Fisher’s exact test, as appropriate. Repeated measures analysis of variance (ANOVA) was utilized to compare the data collected within and between groups at multiple time points. In order to access the effects of gender, and dosage of methoxamine and butorphanol on the incidence of POCD, binary logistic regression analysis was performed. The dependent variable was the occurrence of POCD (yes/no), while the independent variables were gender (male/female) and the medication dosages. Gender was included as a categorical variable (male = 0, female = 1), while the dosages of methoxamine and butorphanol were treated as continuous variables. The results were interpreted using odds ratios (ORs), and statistical significance was determined at *p* < 0.05. All analyses were performed using SPSS. Statistical significance was set as *p* < 0.05.

## 3 Results

### 3.1 Comparison of baseline characteristics and intraoperative data between groups

**Figure 1:**
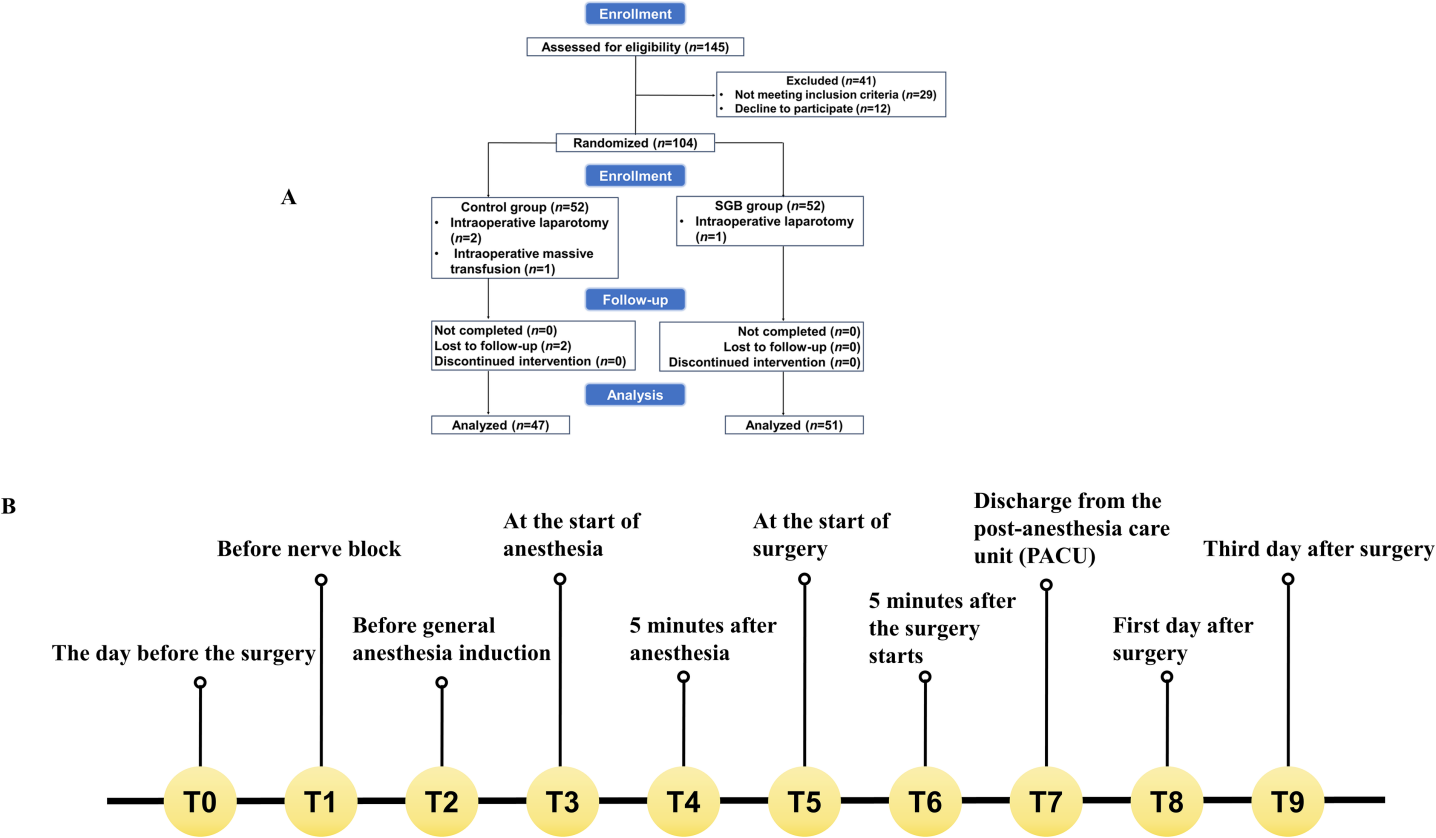
Study flowchart and event timeline. **(A)** The clinical trial flowchart illustrates the enrollment, randomization, allocation to the control and SGB groups, interventions, follow-up, and analysis of participants. **(B)** The event timeline depicts the schedule of interventions and assessments at standardized time points (T0, T1, T2, etc.) throughout the study.

The present study screened 145 older adult patients, who were scheduled for laparoscopic GI surgery between June 2023 and December 2023. Forty-one patients were excluded. Among these patients, 29 patients did not meet the inclusion criteria, and 12 patients declined to participate. Thus, a total of 104 patients remained for the present study. These patients consented to participate, and provided a signed informed consent form. During the study period, six more patients were excluded: two patients were lost to follow-up, three patients required conversion to open surgery, and one patient experienced major intraoperative bleeding that required transfusion of over three units of red blood cells. Therefore, a total of 98 patients were included in the final analysis. The participant flow diagram is detailed in Figure 1.

As demonstrated in Supplementary Table S1, no statistically significant differences were found in the baseline characteristics between the two groups (*p* > 0.05). The characteristics were, as follows: age, gender, BMI, education level, pre-existing comorbidities, lifestyle habits (smoking and drinking), ASA status, type of surgical procedure performed, and the presence or absence of preoperative anemia.

Although the groups were balanced, in terms of baseline characteristics, significant differences in intraoperative management were observed. Specifically, patients in the SG group intraoperatively utilized less butorphanol and more methoxamine, when compared to the control group (*p* < 0.05, Supplementary Table S1). However, no significant between-group differences were found for other intraoperative factors, including the need for blood transfusion, duration of surgery, and total anesthesia time.

### 3.2 SGB improves cognitive performance and reduces POCD incidence

**Figure 2:**
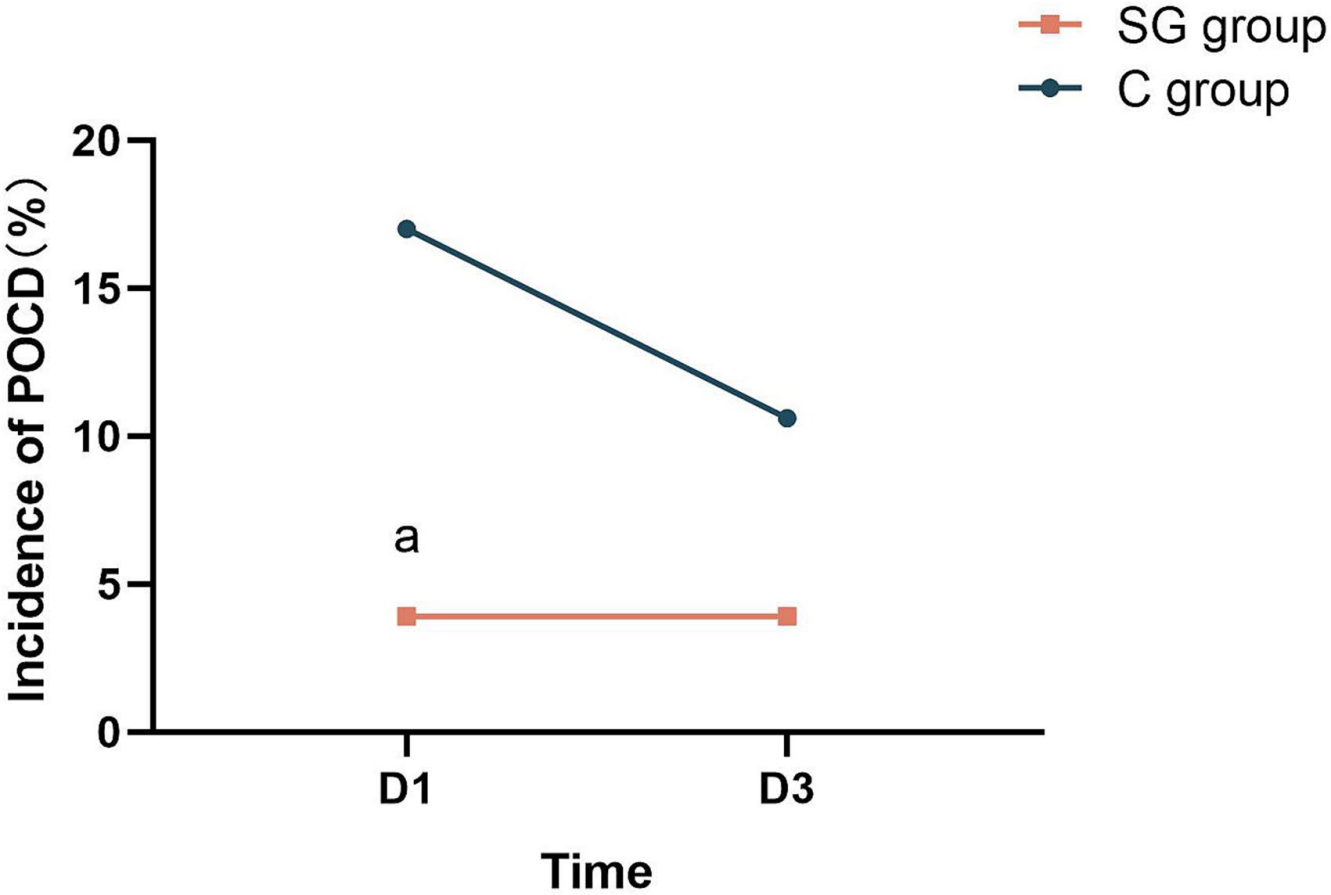
SGB significantly reduces the incidence of POCD in patients. T8, first postoperative day; T9, third postoperative day; POCD, postoperative cognitive dysfunction; ^a^*p* < 0.05 *vs.* the control (C) group.

The present results revealed that SGB positively impacts postoperative cognitive function, and leads to lower incidence of POCD. Specifically, patients in the SG group had significantly higher MMSE scores at T8, when compared to the control group (*p* < 0.05, Table 1). Similarly, the SG group outperformed the control group in terms of the MoCA assessment, at both T8 and T9 (*p* < 0.001–0.05, Table 1), indicating a more sustained cognitive benefit. As determined by the MMSE and MoCA scores, the incidence of POCD on T8 was significantly lower in the SG group, when compared to the control group (*p* < 0.05, Figure 2).

**Table 1:** Comparison of MMSE and MoCA scores at different time points in the two groups of patients.

	Time points	Control group (*n* = 47)	SGB group (*n* = 51)	*Z*	*p*
MMSE	T0	25 (24–26)	26 (25–27)	−1.297	0.195
	T8	24 (23–25)^ab^	24 (24–25)^ab^	−2.215	0.027
	T9	25 (24–26)^b^	25 (24–26)^b^	1.410	0.159
MoCA	T0	27 (27–28)	27 (27–28)	−0.239	0.811
	T8	26 (26–27)^b^	27 (26–27)^ab^	−2.403	0.016
	T9	27 (26–27)	28 (27–28)^a^	−3.692	0.001

Non-parametric tests were used for intergroup comparisons, repeated measures analysis of variance was used for intragroup comparisons, and median (M) and interquartile range (IQR) were used for representation. T0, at one day before surgery; T8, at the first day after surgery; T9, at the third day after surgery; MMSE, Mini-Mental State Examination; MoCA, Montreal Cognitive Assessment; ^a^*p* < 0.05 vs. the control group; ^b^*p* < 0.05 vs. T0.

### 3.3 SGB impacts intraoperative hemodynamics

**Figure 3:**
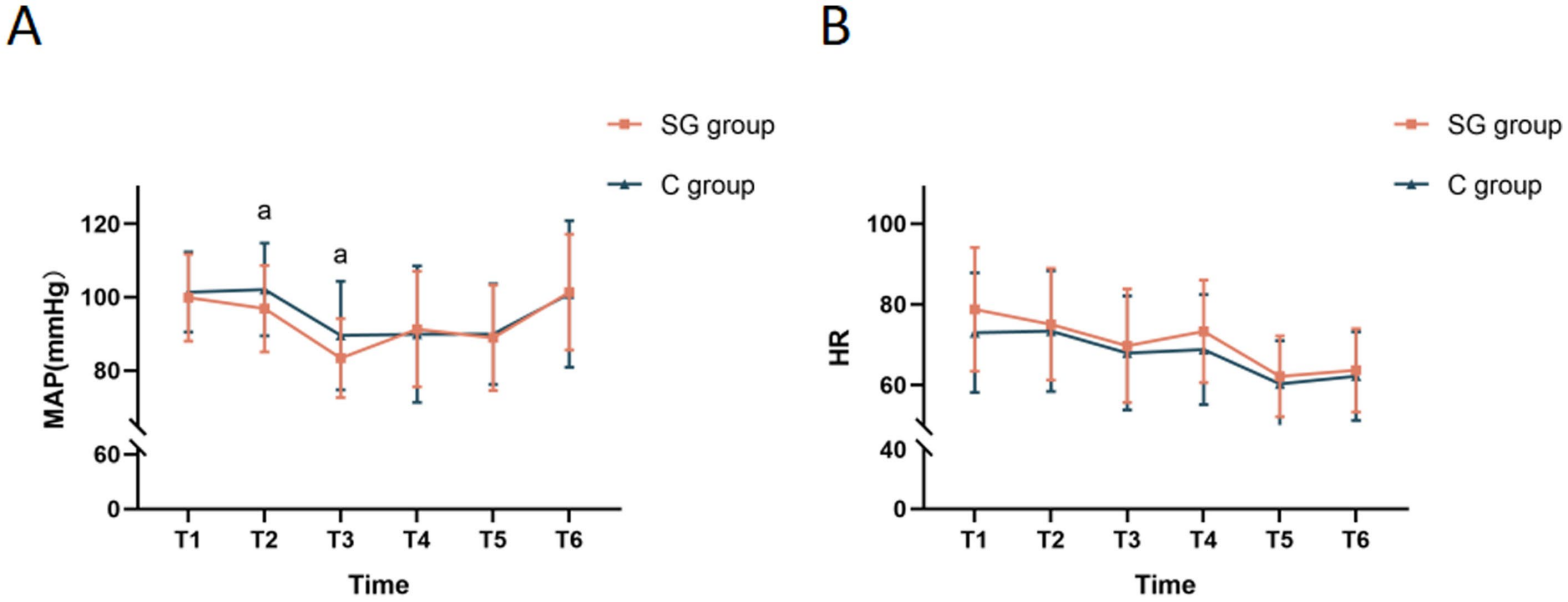
Comparison of MAP and HR at different time points between the two groups. **(A)** Monitored MAP at various time points for both groups. **(B)** Monitored HR at various time points for both groups. The results were presented in mean ± standard deviation (SD), *n* = 47–51. ^a^*p* < 0.05 *vs.* the control group. T1, before the SGB procedure; T2, before anesthesia induction; T3, immediately after anesthesia initiation; T4, at five minutes after anesthesia; T5, at the start of surgery; T6, at five minutes after the start of surgery; MAP, mean arterial pressure; HR, heart rate.

Significant differences in MAP were observed between the SGB and control groups during the intraoperative period. Specifically, the MAP measurements at time points T2 and T3 were significantly lower in the SG group, when compared to the control group (Figure 3A), indicating the potential hypotensive effect of SGB. This finding was particularly interesting, since the controlled hypotension during surgery may have contributed to the improved postoperative cognitive outcomes. However, no significant differences in HR were identified between the two groups throughout the surgery (Figure 3B).

### 3.4 SGB reduces postoperative pain scores without impacting BIS or PONV incidence

The present findings revealed that SGB effectively reduced postoperative pain in the short term, without significantly impacting BIS or the incidence of PONV. As detailed in Table 2, patients who received SGB reported significantly lower pain scores on the NRS at T8, when compared to the control group (*p* < 0.05). However, this analgesic effect was not sustained in the long term, since there were no statistically significant differences in NRS scores between the two groups at T9.

**Table 2:** Comparison of postoperative PONV incidence and NRS scores in the two groups.

	Time points	Control group (*n* = 47)	SGB group (*n* = 51)	*p*
PONV (*n*, %)	T8	7 (14.90)	3 (5.90)	0.255
	T9	1 (2.10)	0 (0)	0.967
NRS (*n*, %)	T8 (1–3)	38 (80.80)	49 (96.10)^a^	0.001
	T8 (4–6)	4 (8.50)	0 (0)^a^	
	T9 (1–3)	41 (87.30)	43 (98.00)	0.845
	T9 (4–6)	1 (2.10)	1 (2.00)	

Qualitative data was presented in frequency (percentage). PONV, postoperative nausea and vomiting; NRS, numeric rating scale; ^a^*p* < 0.05 vs. the control group.

Importantly, there were no significant between-group differences identified in the incidence of PONV at T8 or T9 (Table 2), indicating that SGB did not appear to increase the risk of postoperative nausea and vomiting. Similarly, the SGB administration did not significantly affect BIS at any of the monitored time points (T1-T6, Supplementary Table S2). This suggests that SGB does not interfere with the depth or induction of anesthesia.

### 3.5 SGB modulates inflammatory and oxidative stress markers

**Figure 4:**
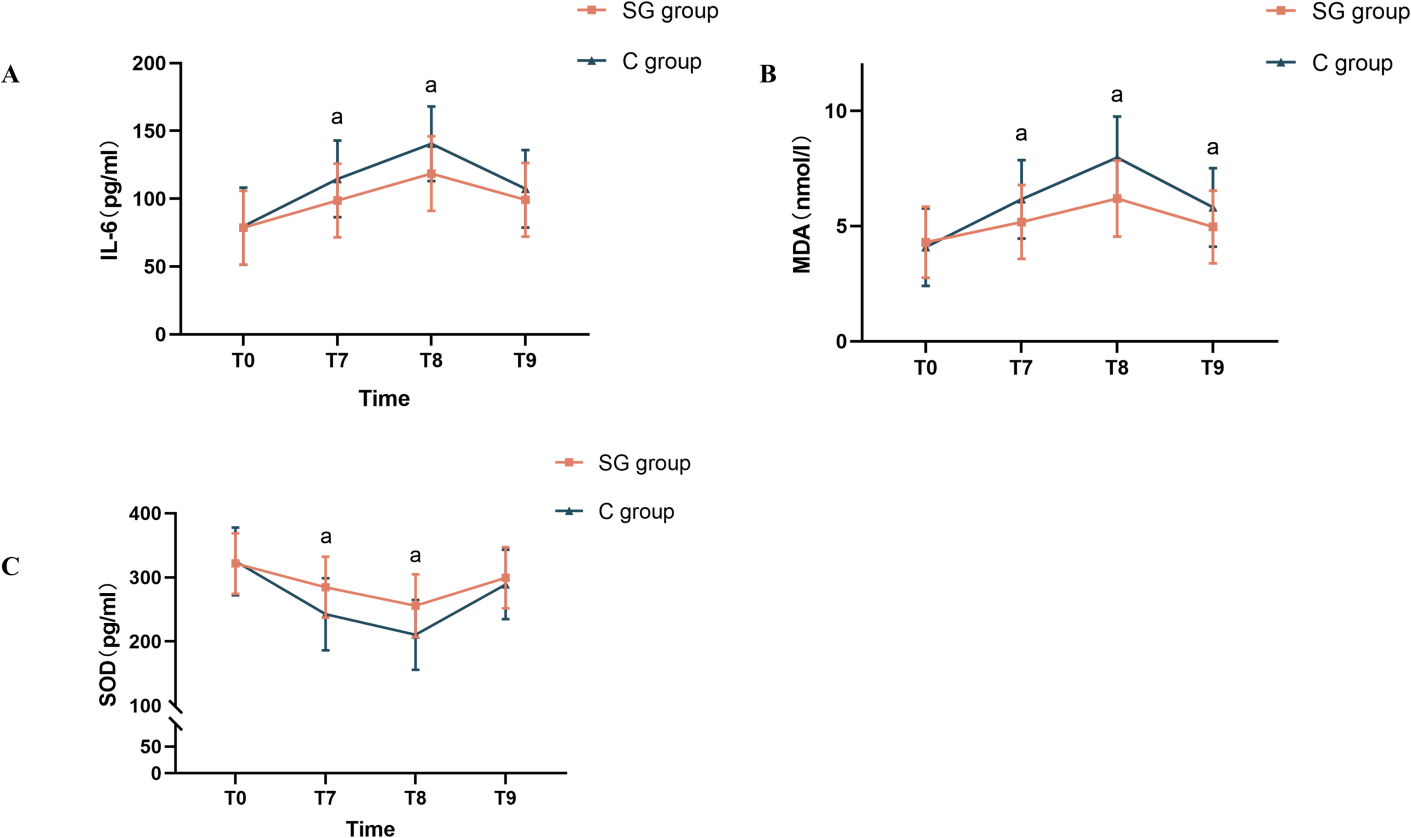
Comparison of peripheral blood levels of IL-6, SOD, and MDA at different time points between the two groups. **(A)** Changes in serum IL-6 levels at various time points in both groups. **(B)** Changes in serum MDA levels at various time points in both groups. **(C)** Changes in serum SOD levels at various time points in both groups. The results were presented in mean ± standard deviation (SD), *n* = 47–51. ^a^*p* < 0.05 *vs.* the control group. T1, before the SGB procedure; T2, before anesthesia induction; T3, immediately after anesthesia initiation; T4, at five minutes after anesthesia; T5, at the start of surgery; T6, at five minutes after the start of surgery; IL-6, interleukin-6; SOD, superoxide dismutase; MDA, malondialdehyde.

The present analysis revealed that SGB significantly modulated the inflammatory and oxidative stress markers in the perioperative period. As shown in Figure 4, statistically significant differences in IL-6, SOD, and MDA levels were observed between the SGB and control groups at time points T7 and T8 (*p* < 0.05). This modulation appeared to be most pronounced earlier in the surgical course, since only the MDA levels remained significantly different between these groups at time point T9 (*p* < 0.05).

Specifically, compared to the control group, patients in the SGB group presented with significantly lower levels of pro-inflammatory cytokine IL-6 and MDA (a marker of lipid peroxidation) at T7 and T8 (*p* < 0.05, Figures 4A,B). Conversely, the antioxidant enzyme SOD was significantly elevated in the SGB group, when compared to the control group, at those same time points (*p* < 0.05, Figure 4C). These findings collectively suggest that SGB effectively reduces both inflammation and oxidative stress in the early perioperative period.

## 4 Discussion

The present study provides evidence that SGB can effectively reduce the incidence of POCD at the first postoperative day in elderly patients who underwent GI surgery. Patients who received SGB presented with significantly higher MMSE and MoCA scores at one day after surgery, when compared to the control group, indicating improved cognitive function. Furthermore, these patients presented with lower serum levels of IL-6 and MDA, along with elevated SOD levels. This suggests that the neuroprotective effects of SGB may be partly due to its ability to attenuate the inflammatory cascade and oxidative stress during the perioperative period (Figure 4).

The reported incidence of POCD considerably varies, which is likely due to the differences in surgical procedures, anesthesia techniques, and assessment tools employed across studies. A recent systematic review reported a POCD incidence that ranged within 2.20–31.50% at one week to three months after non-cardiac surgery, and a POCD incidence that ranged within 11.80–35.70% after cardiac surgery (Arefayne et al., 2023). The present findings for the control group are consistent with this reported range. Notably, the SGB group in the present study had a POCD incidence of 3.90% on both the first and third postoperative days, while the control group presented with a decline in POCD incidence (from 17.00% at postoperative day one to 10.60% at postoperative day three). This contrast further highlights the potential of SGB to promote positive postoperative cognitive outcomes. Although the present study focused on a Chinese patient population, it is crucial to acknowledge that frailty is a significant risk factor for POCD (Tonelli et al., 2021). Interestingly, Chinese elderly patients tend to present with lower levels of frailty, when compared to their counterparts in other low- and middle-income countries, as well as in the United States (Woo et al., 2015). This difference underscores the urgency of addressing and mitigating POCD risk within the Chinese elderly population.

It was estimated that approximately 50% of elderly individuals undergo surgical procedures (Kotekar et al., 2018), with major abdominal surgery, particularly GI surgery, being the commonplace in this population (Kawaguchi et al., 2021). These procedures are frequently associated with POCD and other complications. The present lack of effective treatment and preventative strategies for POCD highlights the need for a deeper understanding of its underlying mechanisms. The present study builds upon existing research, further illuminating the complexities of POCD in elderly surgical patients.

Although the precise mechanisms underlying POCD remains to be fully elucidated, previous studies have noted oxidative stress and neuroinflammation as key contributors, highlighting its significant roles in the development of POCD in elderly patients (Li et al., 2023; He et al., 2024). Surgical trauma initiates a cascade of inflammatory responses, including the surge in IL-6 and tumor necrosis factor-*α* (TNF-α). Elevated levels of these inflammatory cytokines are strongly correlated to cognitive decline (Liu et al., 2023). Alterations in oxidative stress markers, such as the increase in MDA and disrupted activity of antioxidant enzymes, including SOD, are also implicated in the pathophysiology of POCD (Lopez et al., 2020).

Furthermore, aging itself is characterized by increased oxidative stress and chronic, low-grade inflammation, which is partly due to the decline of the innate immune system, and the persistent inflammatory state triggered by endogenous ligands (Lin et al., 2020). Surgery and anesthesia can exacerbate these processes, further increasing circulating immune cells and inflammatory cytokines, such as IL-6, compromising the BBB, and promoting the activation of microglia and astrocytes in the central nervous system. This can lead to neuronal damage through oxidative stress and inflammatory responses (Yang and Chen, 2022; Lu et al., 2023).

IL-6 particularly exhibits neurotoxic properties. Elevated postoperative IL-6 levels have been linked to the development of POCD, with the degree of cognitive decline correlating to postoperative plasma IL-6 concentrations (Taylor et al., 2023). The association between IL-6 and cognitive impairment is supported by animal models, wherein elevated IL-6 levels have been linked to poorer cognitive outcomes (Dong et al., 2022).

Oxidative stress frequently occurs alongside mitochondrial dysfunction. The microglia themselves undergo age-related changes in its mitochondria (Agrawal and Jha, 2020; Mossad et al., 2022). Surgery and anesthesia can further tip the scales toward free radical production (e.g., MDA), leading to the imbalance between free radicals and antioxidants (e.g., SOD) (Senoner et al., 2021). This imbalance can result in oxidative damage to the brain, contributing to cognitive impairment. This notion was substantiated by a study that used an aged rat model of tibial fractures. In that study, the researchers observed that rats that exhibited memory impairment presented with hippocampal lipid peroxidation, and reduced antioxidant enzyme activity (Netto et al., 2018).

The present findings align with these previous observations. Elevated levels of IL-6 and MDA, alongside decreased SOD levels, were observed at the conclusion of surgery, and on the first postoperative day. Importantly, these biomarkers did not return to baseline levels, even on the third postoperative day, indicating the persistent state of oxidative stress and inflammation in the early postoperative period. It is highly plausible that these biochemical changes represent the key driver of postoperative cognitive decline.

**Figure 5:**
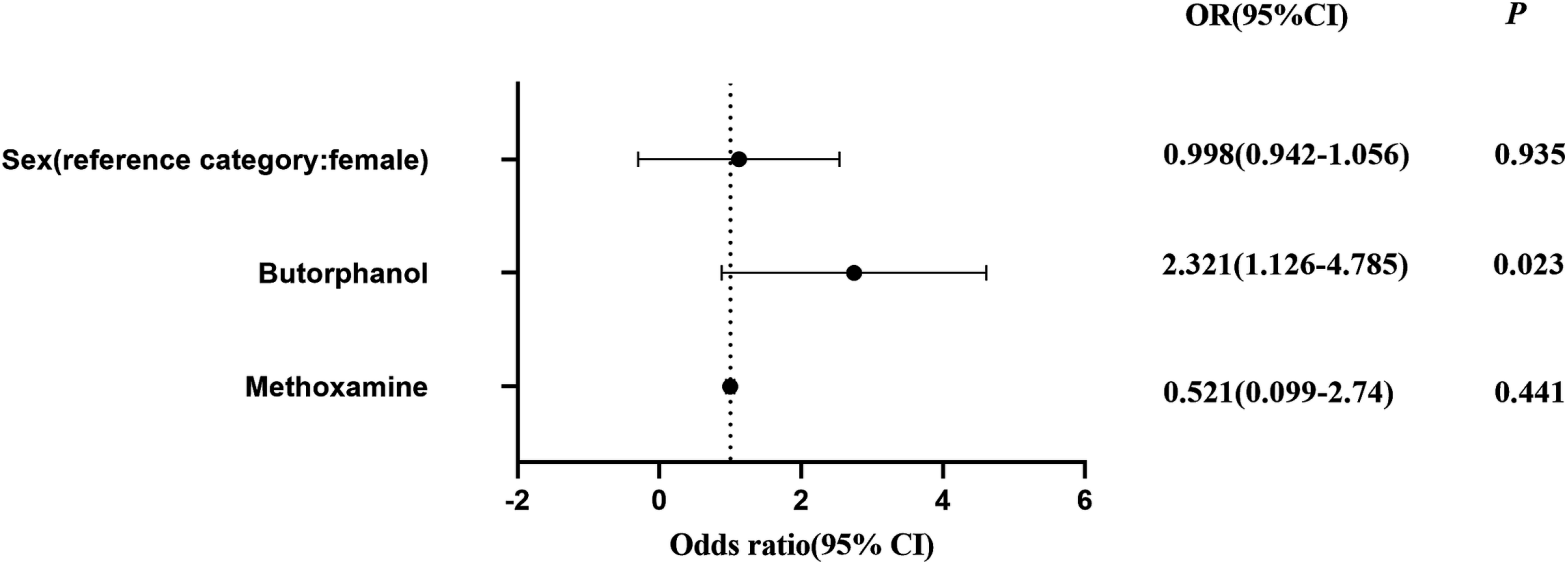
Logistic regression analysis of the effect of SGB on the incidence of POCD before and after adjusting for gender, and dosages of butorphanol and methoxamine. The odds ratios (ORs) with 95% confidence intervals are presented; *p* < 0.05 indicates a statistically significant difference.

In the present study, it was observed that the dosage of butorphanol had a significant impact on the risk of POCD. After adjustment, the dosage of butorphanol was positively correlated to the risk of POCD, indicating that higher doses of butorphanol are associated to greater risk of developing POCD. Specifically, in the regression analysis, after adjusting for other potential confounding factors, the OR for butorphanol dosage was greater than 1, suggesting that the dosage of butorphanol may be an important predictor of POCD occurrence (Figure 5).

This finding is consistent with previous researches that revealed that the use of anesthetic and analgesic drugs is closely associated to risk of POCD. Butorphanol, as a potent analgesic, may influence postoperative cognitive function through its direct effects on the nervous system. Although a positive correlation was identified between butorphanol dosage and the occurrence of POCD, further studies are needed to explore the underlying mechanisms, particularly how butorphanol may alter neurotransmitter levels or impact neural networks in the brain, thereby increasing the risk of cognitive dysfunction, even after controlling for other factors, such as type of surgery and anesthesia method.

Numerous studies have substantiated the beneficial effects of SGB. The stellate ganglion is known to regulate pain, inflammation, and immune dysregulation in the upper body, and exerts indirect control over both the brain and gut, despite its distance from these organs (Lipov et al., 2020; Wen et al., 2020; Deng J. J. et al., 2023). Although the efficacy of SGB in preventing POCD has been less extensively studied, recent research has supported its potential in this domain. Emerging evidence suggests that SGB can enhance learning and memory, while effectively mitigating postoperative cognitive decline (Yu et al., 2023). For instance, SGB administration has been shown to increase SOD levels, and decrease MDA levels, effectively inhibiting hippocampal neuronal ferroptosis, and attenuating brain injury (Zhou et al., 2023). In addition, SGB appears to modulate sympathetic nervous system overactivity, temporarily interrupting existing pathological pathways, and guiding the nervous system toward a new equilibrium. This modulation would ultimately contribute to the suppression of inflammatory and oxidative stress responses (Fischer et al., 2022).

The present findings are consistent with existing research, demonstrating that patients who received SGB had significantly lower postoperative IL-6 and MDA levels, along with increased SOD activity. These results suggest that SGB may exert neuroprotective effects by regulating the inflammatory and oxidative stress pathways, potentially *via* its modulation of the autonomic nervous system. By inhibiting sympathetic nervous system overactivity, SGB may reduce the release of postoperative inflammatory cytokines, and mitigate postoperative stress response, ultimately helping to preserve cognitive function.

Although concerns on the potential complications associated to SGB persist (Rae Olmsted et al., 2020; Wen et al., 2021), the present study did not observe any significant adverse effects, with no statistically significant differences in hemodynamic changes between the two groups. Interestingly, the SGB group required higher doses of methoxamine, but required significantly lower doses of butorphanol tartrate, when compared to the control group. This observation further supports the potential of SGB as a preventative intervention for POCD in elderly patients who undergo GI surgery, potentially leading to improved postoperative recovery, and a reduced need for potent analgesics. Furthermore, although SGB may have a minor impact on blood pressure (Wen et al., 2021), the present findings indicate that this effect is negligible in clinical practice, particularly when weighed against its numerous advantages.

The present study has several limitations that are important to acknowledge. First, the sample size was relatively small and limited to a specific patient population: elderly patients who underwent laparoscopic GI surgery. Therefore, these present findings may not be generalizable to other surgical populations. Second, the present study employed a relatively short follow-up period, restricting the observations to early postoperative cognitive changes. Future research with extended follow-up periods is needed to assess the long-term cognitive outcomes and recovery patterns in this patient population. While this study assessed key pre- and post-operative inflammatory and oxidative stress markers, it did not include intraoperative sampling at specific peri-anesthetic and early surgical time points (T2 ~ T6). Consequently, the immediate perioperative dynamics of these markers remain uncharacterized. Future research with more granular sampling, particularly within the perioperative window, would enable a more comprehensive analysis of real-time changes in inflammatory and oxidative stress. To enhance the robustness and generalizability of these findings, future studies should include larger and more diverse cohorts, which comprise of a broader spectrum of surgical procedures and patient demographics, in order to further validate the efficacy of SGB in preventing postoperative cognitive dysfunction.

## 5 Conclusion

In conclusion, the present results suggest that SGB may represent a promising prophylactic approach for mitigating POCD in elderly patients who undergo GI surgery. Beyond its potential for reducing postoperative cognitive decline, SGB may contribute to improved overall patient outcomes in this population. These present findings offer compelling evidence that SGB, through its modulation of inflammatory and oxidative stress responses, represents a valuable intervention strategy for preserving cognitive function in elderly surgical patients, and warrants further exploration in clinical practice.

## Data Availability

The raw data supporting the conclusions of this article will be made available by the authors, without undue reservation.
