# Supplementary material for: Improved early postoperative cognition in elderly gastrointestinal patients: a randomized controlled trial on the role of ultrasound-guided stellate ganglion block
Source: Front Aging Neurosci. 2025 Feb 11;17:1503314. doi: 10.3389/fnagi.2025.1503314 (PMC11850532; doi:10.3389/fnagi.2025.1503314)
Supplement: Supplementary file 1 [file Data_Sheet_1.DOCX]

**Table S1. Comparison of baseline characteristics and intraoperative conditions between the two groups of patients.**

|  | **Control group (*n*=47)** | **SGB group (*n*=51)** |  | ***p*** |
| --- | --- | --- | --- | --- |
| **Age (years, M[IQR])** | 72 (69-78) | 71 (68-77) |  | 0.770 |
| **Gender (*n*, %)**  **Male** | 30 (63.8) | 34 (66.7) |  | 0.768 |
| **Female** | 17(36.2) | 17(33.3) |  |  |
| **BMI (kg/m²,‾χ±s）** | 23.3 ± 3.6 | 22.7 ± 2.4 |  | 0.379 |
| **Education level (*n*, %)** |  |  |  | 0.916 |
| **Ⅰ (Primary school)** | 26 (55.3) | 26 (51) |  |  |
| **II (Middle school)** | 11 (23.4) | 15 (29.4) |  |  |
| **III (High school)** | 6 (12.8) | 6 (11.8) |  |  |
| **IV (University and above)** | 4 (8.5) | 4 (7.9) |  |  |
| **Preoperative comorbidities (*n*, %)** |  |  |  |  |
| **Hypertension** | 24 (51.1) | 25 (49) |  | 0.840 |
| **Diabetes** | 14 (29.8) | 16 (31.4) |  | 0.865 |
| **Coronary heart disease** | 0 | 5 (9.8) |  | 0.081 |
| **OSAS** | 3 (6.4) | 1 (2) |  | 0.552 |
| **Arrhythmia** | 11 (23.4) | 12 (23.5) |  | 0.988 |
| **Cerebrovascular disease** | 1 (2.1) | 4 (7.8) |  | 0.409 |
| **Long-term smoking (*n*, %)** | 5 (10.6) | 0 |  | 0.053 |
| **Long-term alcohol consumption (*n*, %)** | 5 (10.6) | 1 (2) |  | 0.171 |
| **ASA classification (*n*, %)** |  |  |  | 0.624 |
| **II** | 5 (10.6) | 3 (5.9) |  |  |
| **III** | 42 (89.4) | 48 (94.1) |  |  |
| **Surgical type (*n*, %)** |  |  |  | 0.646 |
| **Gastric surgery** | 9 (19.1) | 14 (27.5) |  |  |
| **Colonic surgery** | 22 (46.8) | 22 (43.1) |  |  |
| **Rectal surgery** | 12(25.5) | 9 (17.6) |  |  |
| **Pancreaticoduodenal surgery** | 4 (8.5) | 6 (11.8) |  |  |
| **Anemia (*n*, %)** | 21 (44.7) | 20 (39.2) |  | 0.584 |
| **Blood transfusion (*n*, %)** | 0 | 4 (7.8) |  | 0.147 |
| **Anesthetic drug dosage (mg, M[IQR])** |  |  | **Difference in medians (95% CI)** |  |
| **Propofol** | 540 (425-680) | 660 (480-760) | 80(0 to 160) | 0.054 |
| **Cisatracurium** | 33 (27-42) | 36 (32-40) | 3(0 to 6) | 0.093 |
| **Butorphanol tartrate** | 3.2 (2.8-4) | 2.6 (2.4-2.8)^a^ | -0.6(-1.1 to -0.4) | 0.001 |
| **Methoxamine (mg, M[IQR])** | 5 (3-8) | 8 (5-17)^a^ | 4(2 to 7) | 0.001 |
| **Surgical duration (min, M[IQR])** | 175（132-247） | 205(175-240) | 23(-5 to 50) | 0.259 |
| **Anesthetic duration (min, M[IQR])** | 190(156-263) | 215(185-253) | 20(-6 to 45) | 0.322 |

**Note:** Data that conformed to the normal distribution was presented in mean ± standard deviation (SD), and data that did not conform to the normal distribution was presented in median (M) and interquartile range (IQR); Qualitative data was presented in frequency (percentage). CI, confidence interval;ASA, American Society of Anesthesiologists; BMI, body mass index; OSAS, obstructive sleep apnea syndrome; ^a^*p*<0.05 *vs.* the control group.

**Table S2. Comparison of BIS in the two groups.**

| **Time points** | **Control group (*n*=47)** | **SGB group (*n*=51)** | ***p*** |
| --- | --- | --- | --- |
| **T1** | 94.1 ± 3.9 | 95.7 ± 4.4 | 0.071 |
| **T2** | 51.7 ± 12.9^b^ | 49.0 ± 11.8^b^ | 0.291 |
| **T3** | 53.4 ± 7.8^b^ | 51.7 ± 9.7^b^ | 0.318 |
| **T4** | 52.9 ± 7.4^b^ | 53.5 ± 8.9^b^ | 0.722 |
| **T5** | 51.7 ± 6.7^b^ | 50.7 ± 7.6^b^ | 0.501 |
| **T1** | 94.1 ± 3.9 | 95.7 ± 4.4 | 0.071 |

**Note:** Quantitative data was presented in mean ± standard deviation (SD). T1, before anesthesia induction; T2, immediately after anesthesia induction; T3, at five minutes after anesthesia; T4, at the start of surgery; T5, at five minutes after the start of surgery; ^b^*p*<0.05 *vs.* T1.

**Table S2. Comparison of BIS in the two groups.**

| **Time points** | **Control group (*n*=47)** | **SGB group (*n*=51)** | ***p*** |
| --- | --- | --- | --- |
| **T1** | 94.1 ± 3.9 | 95.7 ± 4.4 | 0.071 |
| **T2** | 51.7 ± 12.9^b^ | 49.0 ± 11.8^b^ | 0.291 |
| **T3** | 53.4 ± 7.8^b^ | 51.7 ± 9.7^b^ | 0.318 |
| **T4** | 52.9 ± 7.4^b^ | 53.5 ± 8.9^b^ | 0.722 |
| **T5** | 51.7 ± 6.7^b^ | 50.7 ± 7.6^b^ | 0.501 |
| **T1** | 94.1 ± 3.9 | 95.7 ± 4.4 | 0.071 |

**Note:** Quantitative data was presented in mean ± standard deviation (SD). T1, before anesthesia induction; T2, immediately after anesthesia induction; T3, at five minutes after anesthesia; T4, at the start of surgery; T5, at five minutes after the start of surgery; ^b^*p*<0.05 *vs.* T1.
